# Revisiting *Wolbachia* detections: Old and new issues in *Aedes aegypti* mosquitoes and other insects

**DOI:** 10.1002/ece3.11670

**Published:** 2024-07-02

**Authors:** Perran A. Ross, Ary A. Hoffmann

**Affiliations:** ^1^ Pest and Environmental Adaptation Research Group, School of BioSciences, Bio21 Molecular Science and Biotechnology Institute The University of Melbourne Melbourne Victoria Australia

**Keywords:** 16S sequencing, *Aedes aegypti*, mosquito, *Wolbachia*

## Abstract

*Wolbachia* continue to be reported in species previously thought to lack them, particularly *Aedes aegypti* mosquitoes. The presence of *Wolbachia* in this arbovirus vector is considered important because releases of mosquitoes with transinfected *Wolbachia* are being used around the world to suppress pathogen transmission and these efforts depend on a lack of *Wolbachia* in natural populations of this species. We previously assessed papers reporting *Wolbachia* in natural populations of *Ae*. *aegypti* and found little evidence that seemed convincing. However, since our review, more and more papers are emerging on *Wolbachia* detections in this species. Our purpose here is to evaluate these papers within the context of criteria we previously established but also new criteria that include the absence of releases of transinfections within the local areas being sampled which has contaminated natural populations in at least one case where novel detections have been reported. We also address the broader issue of *Wolbachia* detection in other insects where similar issues may arise which can affect overall estimates of this endosymbiont more generally. We note continuing shortcomings in papers purporting to find natural *Wolbachia* in *Ae*. *aegypti* which are applicable to other insects as well.

## Graphical abstract

*Wolbachia* continue to be reported in species previously thought to lack them, particularly *Aedes aegypti* mosquitoes. Here we review reports of natural *Wolbachia* in this species and highlight common issues with molecular detection. Our results are relevant to the detection of endosymbionts in insects and the ongoing releases of *Wolbachia* mosquitoes for arbovirus control.

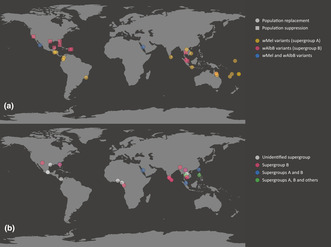

## 1 INTRODUCTION

The maternally inherited endosymbiotic bacterium, *Wolbachia pipientis*, is becoming an important tool in reducing the transmission of dengue and other viral pathogens transmitted by mosquitoes (Hoffmann et al., 2024; Indriani et al., 2023; Ryan et al., 2019). The endosymbiont can have two important impacts on mosquitoes that influence pathogen transmission, the first being the ability to cause cytoplasmic incompatibility (CI) in hosts that causes females lacking *Wolbachia* to become effectively sterile when mated with males carrying *Wolbachia* (Hoffmann & Turelli, 1997), and the second being the ability of the endosymbiont to directly impact through multiple mechanisms the ability of the mosquitoes to pass arboviruses picked up from a person to another individual (Ant et al., 2018; Moreira et al., 2009). CI is an essential component of the *Wolbachia* incompatible insect technique (IIT) applied for suppressing native mosquito populations, where released male *Wolbachia* carriers mate with native females to eventually reduce the size of mosquito populations. This approach is often accompanied by an additional radiation dose applied to released mosquitoes to ensure that any females carrying *Wolbachia* released due to inaccurate sexing do not become established (Zheng et al., 2019). CI is also an essential component of the replacement technique where releases of males and females carrying *Wolbachia* can result in the replacement of the natural mosquito population with those carrying the target *Wolbachia* strain capable of suppressing pathogen transmission (Hoffmann et al., 2011).

For both the population suppression and replacement approaches to work, it is essential that the targeted mosquito populations do not carry *Wolbachia* strains that prevent the expression of CI. For this reason, samples of target populations are typically screened prior to the initiation of releases. Any detection of natural *Wolbachia* should be followed up by crossing experiments to establish patterns of cross incompatibility which can be particularly complex in mosquito species like *Culex pipiens* (Atyame et al., 2011; Duron et al., 2006). This might lead to practitioners selecting different strains of *Wolbachia* for releases in a specific target area.

*Aedes aegypti* mosquitoes have been targeted by both the replacement approach and the suppression approach (Consortium & Ching, 2021; Crawford et al., 2020; Hoffmann et al., 2011; Indriani et al., 2023; Nazni et al., 2019). This species is the main vector of dengue virus in tropical areas and in the past has been considered as lacking *Wolbachia* (Gloria‐Soria et al., 2018). In addition, suppression releases have targeted *Aedes albopictus* (Zheng et al., 2019) which is often naturally infected by two *Wolbachia* strains, *w*AlbA and *w*AlbB, and is considered a poorer vector of dengue (Lambrechts et al., 2010) but not other viruses such as chikungunya (Vega‐Rúa et al., 2014). As *Wolbachia* releases have expanded to new countries, researchers have become interested in screening local *Aedes* species for *Wolbachia*, focussing particularly on *Ae*. *aegypti*.

In a previous report on *Wolbachia* detections in *Ae*. *aegypti* (Ross, Callahan, et al., 2020), we identified 8 studies purporting to detect natural infections. Unfortunately, there are issues involved in accurate detection and characterization of natural *Wolbachia* in mosquitoes and other insects which requires follow up work to confirm an infection and characterize it phenotypically. Of these studies, only two established lab populations to confirm the infection in lab stocks (Balaji et al., 2019; Kulkarni et al., 2019). We found at least one case where the infection then could not be confirmed from those stocks (Kulkarni et al., 2019; Ross, Callahan, et al., 2020). The main reason for this note is to reiterate issues with *Wolbachia* detection as more and more papers continue to report *Wolbachia* infections in *Ae*. *aegypti* (Table 1) and other species. We discuss potential explanations for false positive detections and highlight cases where detections likely reflect released transinfections rather than natural infections.

**TABLE 1:** Detections of purportedly natural *Wolbachia* strains in *Aedes aegypti* mosquitoes.^a^

Location	Collection dates	Evidence	Percent positive (n tested)	Supergroups	References
Jacksonville, Florida	July 2014	16S rRNA sequencing, MLST detection	Not specified	A, B	Coon et al. (2016)
Kuala Lumpur, Malaysia	Not specified	*wsp* detection	25% (16)	Unidentified	Teo et al. (2017)
Nakhon Nayok, Thailand	2008	16S and 18S rRNA sequencing	Not specified	C, others	Thongsripong et al. (2018)
Houston, Texas, USA	Not specified	16S rRNA sequencing	Not specified	Unidentified	Hegde et al. (2018)
Tamil Nadu, India	August 2015	16S rRNA, *wsp*, MLST detection Electron microscopy qPCR across developmental stages and tissues Antibiotic removal	Not specified	B	Balaji et al. (2019)
New Mexico and Florida, USA	2016, 2017	*gatB*, *ftsZ* detection, LAMP detection Maternal transmission	44.8% (194)	B	Kulkarni et al. (2019)
Manila, Philippines	May 2014–January 2015	Wsp, 16S rDNA detection	11.9% (672)	A, B, C, D, J	Carvajal et al. (2019)
Panama	Not specified	16S rRNA sequencing	0.2% (490)	Unidentified	Bennett et al. (2019)
Selangor, Malaysia	2013–2019	*wsp*, 16S rRNA detection	100% for *wsp*, 0% for 16S rRNA (2)	Unidentified	Wong et al. (2020)
Manila, Phillipines	June–September 2017	wsp, 16S rRNA sequencing	0.84% (*n* = 359)	A	Regilme et al. (2021)
Lopé village, Gabon	November–December 2017 and April–May 2018	16S rRNA sequencing	7.3% (55)	B	Zouache et al. (2022)
Tamil Nadu, India	March–May 2019	16S rDNA sequencing	66.7% (3) of pools	B	Kumar et al. (2022)
Nakhon Ratchasima, Thailand	August 2017–November 2018	*wsp*, 16S rDNA, 28 s rDNA sequencing	9.1% (11) of pools	Unidentified	Surasiang et al. (2022)
Yunnan Province, China	October–November 2018	*wsp* sequencing	5% (480)	A, B	Zhang et al. (2022)
Mueang Khon Kaen, Thailand	Not specified	16S rRNA sequencing	Not specified	Unidentified	Rodpai et al. (2023)
Jinghong City, China	October–December 2018	16S rRNA sequencing	100% (19) of pools	B	Li et al. (2023)
Kaohsiung City, Taiwan	Not specified	*wsp* detection with nested PCR	3.3% (665)	A, B	Chao and Shih (2023)
Northeast India	2018–2019	16S rRNA sequencing	38.3% (115) of pools	B	Vinayagam et al. (2023)
Jeddah, Saudi Arabia	Not specified	*wsp*, 16S rRNA sequencing, Detection in laboratory colony	23.1% for 16S rRNA, 0% for wsp (13)	A, B	Somia et al. (2023)
Manila, Phillipines	May 2014–January 2015	ddRADseq, wsp, 16S rRNA detection, qPCR	39.2% for wsp, 22.6% for 16S rRNA (217)	A, B, D	Muharromah et al. (2023)
Southern Benin	April–October 2021	16S rDNA detection	47% (15) of pools	Unidentified	Ateutchia‐Ngouanet et al. (2024)
Manila, Phillipines	May 2014–January 2015	wsp detection, 16S rRNA sequencing, qPCR	40.1% for 16S rRNA, 62.2% for *wsp* (429)	A, B	Reyes et al. (2024)
Bioko Island, Equatorial Guinea	February 2020–August 2021	*hscA* detection with qPCR	20% (10) of pools	Unidentified	Giger et al. (2024)
Selangor, Malaysia	November 2022–February 2023	*wsp* sequencing	38.6% (70)	A, B	Roslan et al. (2024)
Morelos, Mexico	June–July 2016	16S sequencing	21.4% (14) of pools	Unidentified	Hernández et al. (2024)
Sri Lanka	October 2014–June 2022	*wsp*, 16S, MLST sequencing	3.35% (507)	B	Wijegunawardana et al. (2024)

^a^ Note that first 8 cases listed here were considered in Ross, Callahan, et al. (2020); the other cases are new studies.

## 2 CHALLENGES IN NEW STUDIES

We have identified 26 studies purporting to detect natural *Wolbachia* in *Ae*. *aegypti* in field populations (Table 1) and two others involving laboratory experiments based on one of these natural infections (Balaji et al., 2021; Balaji & Prabagaran, 2022). Twenty‐one of these studies specifically claimed to detect natural *Wolbachia* infections as opposed to DNA sequences. Recent *Wolbachia* survey studies often cite previous detections uncritically as justification for conducting their own study, or as being in support of their own results, but continue to ignore issues raised previously. A challenge is that molecular approaches for detecting *Wolbachia* and other endosymbionts have their limitations. Molecular detection is often focused on one approach such as 16S rRNA which may detect *Wolbachia* among a community of other bacteria (Rodpai et al., 2023). This approach is prone to contamination, particularly when pooled samples are used or when a lab undertakes work on other species which may have a high abundance of *Wolbachia*. It also cannot readily be used to quantify endosymbiont densities, given that 16S primers may preferentially amplify some groups which depends on factors like primer efficiency and copy number (Větrovský & Baldrian, 2013).

In studies where multiple molecular markers are employed, these tend to give inconsistent patterns of *Wolbachia* presence (e.g. *wsp* + 16S rRNA comparisons) with one approach performing better in one study but the reverse occurring in another study (Somia et al., 2023; Wong et al., 2020). The incidence of *Wolbachia* detected is often very low (e.g. 3.3% in Taiwan, (Chao & Shih, 2023); 5% Yunnan, China, (Zhang et al., 2022), 7% in Gabon (Zouache et al., 2022)) or cannot be estimated due to the use of pooled data (Vinayagam et al., 2023). Low frequencies are not expected to occur in populations with stable, maternally transmitted *Wolbachia*, and may reflect environmental contamination unless there is a clear geographic pattern. The strains detected often match existing strains being released (Somia et al., 2023) or strains present in related species (Chao & Shih, 2023; Zhang et al., 2022). In one example from northeastern India, only *w*AlbB *Wolbachia* was detected in sympatric *Ae*. *albopictus* and *Ae*. *aegypti* (Vinayagam et al., 2023), whereas the former species is typically double infected with *w*AlbB and *w*AlbA (Yang et al., 2022).

On the other hand, multiple *Wolbachia* types have also purported to have been detected in some population samples. *Aedes aegypti* from Manila were considered infected by at least 4 different *Wolbachia* including strains related to those from *Drosophila melanogaster*, *Culex quinquefasciatus* and *Brugia malayi*, although some of these appeared to be rare based on read numbers (Muharromah et al., 2023). This is an unusually high diversity of *Wolbachia* given that interactions among *Wolbachia* strains based on host effects associated with the *Wolbachia* typically drive some *Wolbachia* out of populations as well documented in *Drosophila* (Kriesner et al., 2013). The presence of multiple *Wolbachia* strains was supported by additional work using different locally developed primers for common markers (Reyes et al., 2024), with a novel low‐density strain being detected. However, it is worth noting that all three molecular papers now developed from Manila (including Carvajal et al., 2019) have used the same original *Ae*. *aegypti* material. We find it surprising that new material was not considered to check for contamination in this instance.

At minimum, we recommend that any molecular detections should be followed up by qPCR on individuals with *Wolbachia*‐specific primers (e.g. *wsp*, *ftz*) and host genes included as controls. Hosts should also be accurately identified such as using COI or ITS2 barcodes. qPCR or digital PCR methods are important in quantifying levels of infection, although read depth has also been successfully used (Muharromah et al., 2023). Where *Wolbachia* levels are particularly low such as reflected in high Ct or Cp values or low read numbers, or can only be detected through nested PCR, there should be particular concern about possible contamination from other biological sources in a laboratory. Some studies in *Ae*. *aegypti* (Kumar et al., 2022; Zouache et al., 2022) and other mosquitoes (Sawadogo et al., 2022) do take a cautious approach when interpreting low frequencies or densities and acknowledge potential sources of contamination. However, the majority of studies listed in Table 1 that claim to have detected a natural *Wolbachia* infection do not acknowledge potential sources of contamination (17/21) or do not specify the need for further validation of these natural infections (15/21).

Since our previous review, there remains a lack of attempts to set up laboratory lines of *Ae*. *aegypti* for detailed evaluations, unlike other systems such as *Anopheles* mosquitoes where the presence of natural *Wolbachia* was previously in doubt (Walker et al., 2021). Establishing laboratory lines of natural *Wolbachia* strains in *Ae*. *aegypti* should be relatively simple given the high frequency of *Wolbachia* apparently present in many populations (Table 1) and the ease at which this species can be reared and tested in the laboratory. If a laboratory stock is available, it is possible to undertake additional experiments to confirm the impact of *Wolbachia* on CI and also confirm the mode of inheritance as being maternal (Ross, Callahan, et al., 2020). There are cases of *Wolbachia* DNA being incorporated into host nuclear DNA (Brelsfoard et al., 2014; Nikoh et al., 2008) which then leads to nuclear rather than maternal inheritance being exhibited by the markers. CI experiments can also test whether any detected natural infection might interfere with replacement by a different *Wolbachia* or IIT based suppression. Lab stocks can be used to undertake further characterization of *Wolbachia* in hosts, such as through fluorescence *in situ* hybridization (FISH) (Czarnetzki & Tebbe, 2004). In fact, laboratory stocks are essential to assess the concerns often used as justification for molecular screening of *Aedes* species.

Balaji and Prabagaran (2022) have now performed additional experiments involving the laboratory population established by Balaji et al. (2019) to further characterize its phenotypic effects including CI. They show that the purported strain *w*AegB does not cause detectable CI, has no significant effect on fitness and does not provide protection against three bacterial pathogens (Balaji & Prabagaran, 2022). While this laboratory population has been confirmed to be positive for *Wolbachia* though PCR (Balaji & Prabagaran, 2022) and 16S rRNA sequencing (Balaji et al., 2021), there has been no further validation beyond molecular detection since the original study (Balaji et al., 2019). Given its close similarity to *w*AlbB in *Ae*. *albopictus* we would expect it to cause CI or at least influence crossing patterns with this strain, however our attempts to contact the authors to perform an independent evaluation and test crossing patterns with *Ae*. *aegypti* transinfections have been unsuccessful.

It is possible that the low detections of some *Wolbachia* strains represent interspecific interactions, notably between (uninfected) *Ae*. *aegypti* and (naturally infected) *Ae*. *albopictus*. Although there is some variation among populations, *Ae*. *albopictus* females are typically infected by both *w*AlbB and *w*AlbA, with males tending to have a lower infection rate of *w*AlbA (Yang et al., 2022). Interspecific matings between male *Ae*. *albopictus* and female *Ae*. *aegypti* occur at a low frequency in nature (Bargielowski & Lounibos, 2014; Tripet et al., 2011) and could result in *Wolbachia* being detected in *Ae*. *aegypti* females even if the host does not transmit the *Wolbachia*. Previous mating experiments (Ross, Axford, et al., 2020) indicate that *Wolbachia* can be detected in uninfected females after mating with an infected male although this effect was evident in *w*MelPop and *w*Mel (a supergroup A infection like *w*AlbA from *Ae*. *albopictus*) rather than in the *w*AlbB strain tested in that paper However, *w*AlbB infections can have substantial genomic variation (Martinez et al., 2022) that may influence their detectability through PCR.

It is also possible (particularly for larval samples) that detections represent *Wolbachia* from other parasites such as nematodes. This is acknowledged in some papers (Thongsripong et al., 2018; Zouache et al., 2022) and could account for low level detections of *Wolbachia*. Detections can reflect extremely low levels of *Wolbachia* that can also be quite diverse, which would seem to suggest other organisms or contaminants, particularly in pooled data. For instance, RNA sequencing of pooled adult mosquito samples from Yunnan indicated a very low density of *Wolbachia* in all adult pools with RPM at 1/40th the level recorded for *Ae*. *albopictus* (Li et al., 2023), whereas qPCR screening indicates higher *Wolbachia* titres of transinfections in *Ae*. *aegypti* (c.f. Yang et al. (2022) and Ross et al. (2023)). The *Wolbachia* diversity based on 16S rRNA sequencing was also incredibly high in contrast to *Wolbachia* from other mosquitoes (Li et al., 2023).

**FIGURE 1:**
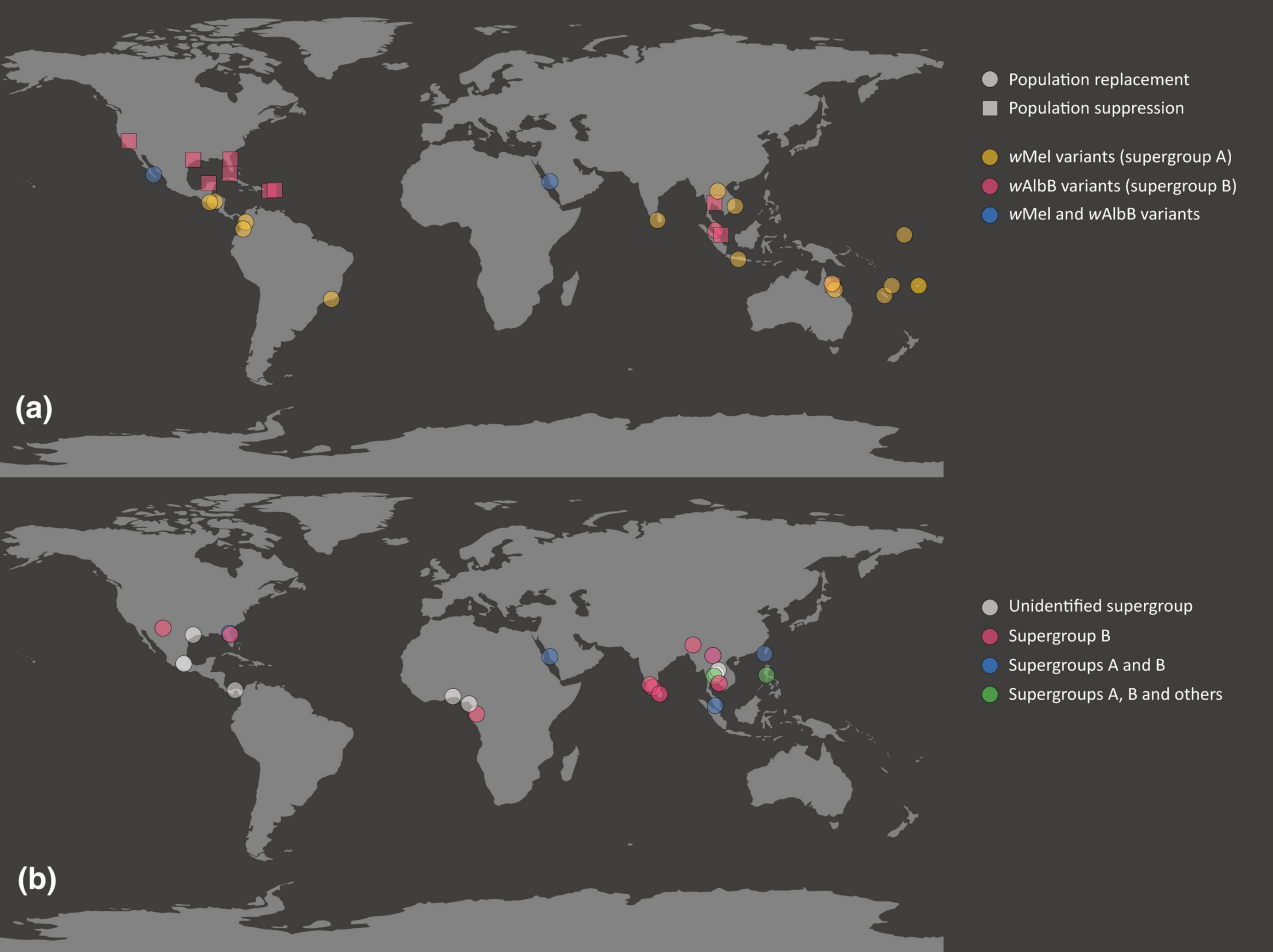
Maps of *Wolbachia* transinfection releases in *Aedes aegypti* (a) and detections of natural *Wolbachia* strains in *Aedes aegypti* (b). Data for transinfection releases were collated from published studies, press releases and personal communications. A list of sources for transinfection releases (a) is provided in Table S1. A list of studies detecting natural *Wolbachia* strains in *Ae*. *aegypti* (b) is provided in Table 1.

In addition, as releases aimed at replacement and suppression continue to expand in countries and around the world (Figure 1), there is an increasing risk of interpreting detected *Wolbachia* as being natural rather than being associated with a release stock. An example of this is Somia et al. (2023) who detect two “natural” infections of *Wolbachia* in Jeddah, Saudi Arabia. *Wolbachia* releases have taken place in Jeddah following a detailed characterization of *w*MelM and *w*AlbB *Wolbachia* strains for release (Ross et al., 2023) and preparatory work at sites (Endersby‐Harshman et al., 2021; Pagendam et al., 2022). With two strains being released, it is not surprising that the authors identified two clades of *Wolbachia* in Jeddah. The authors do not discuss this possibility although they have previously published experimental work on *Wolbachia* field samples that they acknowledge as coming from releases (Algamdi et al., 2023). Other detections of natural *Wolbachia* have also occurred in release areas such as in Selangor, Malaysia (Roslan et al., 2024; Wong et al., 2020) where *Wolbachia* releases were started some time ago (Nazni et al., 2019).

As more *Wolbachia* genomes are introduced into *Ae*. *aegypti* for suppression or replacement (Liu et al., 2022; Sarwar et al., 2022), any new detections of *Wolbachia* should ideally be characterized at this detailed level rather than relying on MLST markers to define strains. While the MLST system has been useful in the past, we are now at the stage where multiple *Wolbachia* variants within the *w*AlbB and *w*Mel strains are being developed and released, reinforcing the usefulness of more detailed genomic comparisons. Full genome analysis also allows for dynamic changes in *Wolbachia* to be tracked across time following releases and (in the case of natural infections) can provide historical information on past *Wolbachia* invasions and dynamics (Gu et al., 2022).

The large number of reports should not be interpreted as robust evidence for the presence of *Wolbachia* in natural *Ae*. *aegypti* populations. We acknowledge that some *Wolbachia* detections are a minor part of a paper aimed at other issues such as overall analysis of microbiota across breeding sites (Zouache et al., 2022) or an assessment of RNA virus diversity (Li et al., 2023). However, these detections should still be interpreted in the context of other potential sources of *Wolbachia* unless additional work is undertaken to confirm stable maternal transmission.

We advocate for researchers to follow the guidelines we developed in our earlier paper (fig 2, Ross, Callahan, et al., 2020) when establishing whether natural *Wolbachia* detections represent heritable endosymbionts. At minimum, laboratory populations should be established and screened with PCR to rule out environmental and non‐heritable sources of *Wolbachia*. If the resources are available, FISH can provide strong evidence of infection and useful information on its distribution within host tissues. While it might be easy to carry out a broad molecular screen for endosymbionts, any findings remain unconvincing until such additional work is carried out.

## AUTHOR CONTRIBUTIONS

**Perran A. Ross:** Conceptualization (equal); writing – original draft (equal); writing – review and editing (equal). **Ary A. Hoffmann:** Conceptualization (equal); writing – original draft (equal); writing – review and editing (equal).

## CONFLICT OF INTEREST STATEMENT

The authors declare that there are no conflicts of interest.

## Supporting information


Table S1.

## Data Availability

All data are contained within the manuscript and its Supporting Information files.

## References

[ece311670-bib-0001] Algamdi, A. G. , Shaher, F. M. , & Mahyoub, J. A. (2023). Biological comparative study between *Wolbachia*‐infected *Aedes aegypti* mosquito and *Wolbachia*‐uninfected strain, Jeddah city, Saudi Arabia. Saudi Journal of Biological Sciences, 30(3), 103581. 10.1016/j.sjbs.2023.103581 36844640 PMC9943864

[ece311670-bib-0002] Ant, T. H. , Herd, C. S. , Geoghegan, V. , Hoffmann, A. A. , & Sinkins, S. P. (2018). The *Wolbachia* strain *w*Au provides highly efficient virus transmission blocking in *Aedes aegypti* . PLoS Pathogens, 14(1), e1006815. 29370307 10.1371/journal.ppat.1006815 PMC5784998

[ece311670-bib-0003] Ateutchia‐Ngouanet, S. , Nanfack‐Minkeu, F. , Mavridis, K. , Wanji, S. , Demanou, M. , Vontas, J. , & Djouaka, R. (2024). Monitoring *Aedes* populations for arboviruses, *Wolbachia*, insecticide resistance and its mechanisms in various agroecosystems in Benin. Acta Tropica, 253, 107178. 10.1016/j.actatropica.2024.107178 38461924

[ece311670-bib-0004] Atyame, C. M. , Delsuc, F. , Pasteur, N. , Weill, M. , & Duron, O. (2011). Diversification of *Wolbachia* endosymbiont in the *Culex pipiens* mosquito. Molecular Biology and Evolution, 28(10), 2761–2772. 10.1093/molbev/msr083 21515811

[ece311670-bib-0005] Balaji, S. , Deepthi, K. N. G. , & Prabagaran, S. R. (2021). Native *Wolbachia* influence bacterial composition in the major vector mosquito *Aedes aegypti* . Archives of Microbiology, 203(8), 5225–5240. 34351459 10.1007/s00203-021-02506-0

[ece311670-bib-0006] Balaji, S. , Jayachandran, S. , & Prabagaran, S. R. (2019). Evidence for the natural occurrence of *Wolbachia* in *Aedes aegypti* mosquitoes. FEMS Microbiology Letters, 366(6), fnz055. 30869785 10.1093/femsle/fnz055

[ece311670-bib-0007] Balaji, S. , & Prabagaran, S. R. (2022). Impact of native *Wolbachia* on reproductive fitness and bacterial pathogens in *Aedes aegypti* mosquitoes. International Journal of Tropical Insect Science, 42(1), 965–975.

[ece311670-bib-0008] Bargielowski, I. , & Lounibos, L. (2014). Rapid evolution of reduced receptivity to interspecific mating in the dengue vector *Aedes aegypti* in response to satyrization by invasive *Aedes albopictus* . Evolutionary Ecology, 28, 193–203. 24563572 10.1007/s10682-013-9669-4 PMC3927939

[ece311670-bib-0009] Bennett, K. L. , Gómez‐Martínez, C. , Chin, Y. , Saltonstall, K. , McMillan, W. O. , Rovira, J. R. , & Loaiza, J. R. (2019). Dynamics and diversity of bacteria associated with the disease vectors *Aedes aegypti* and *Aedes albopictus* . Scientific Reports, 9(1), 12160. 31434963 10.1038/s41598-019-48414-8 PMC6704126

[ece311670-bib-0010] Brelsfoard, C. , Tsiamis, G. , Falchetto, M. , Gomulski, L. M. , Telleria, E. , Alam, U. , Doudoumis, V. , Scolari, F. , Benoit, J. B. , & Swain, M. (2014). Presence of extensive *Wolbachia* symbiont insertions discovered in the genome of its host *Glossina morsitans morsitans* . PLoS Neglected Tropical Diseases, 8(4), e2728. 24763283 10.1371/journal.pntd.0002728 PMC3998919

[ece311670-bib-0011] Carvajal, T. M. , Hashimoto, K. , Harnandika, R. K. , Amalin, D. M. , & Watanabe, K. (2019). Detection of *Wolbachia* in field‐collected *Aedes aegypti* mosquitoes in metropolitan Manila, Philippines. Parasites & Vectors, 12, 1–9. 31340862 10.1186/s13071-019-3629-y PMC6657204

[ece311670-bib-0012] Chao, L.‐L. , & Shih, C.‐M. (2023). First detection and genetic identification of *Wolbachia* endosymbiont in field‐caught *Aedes aegypti* (Diptera: Culicidae) mosquitoes collected from southern Taiwan. Microorganisms, 11(8), 1911. 37630471 10.3390/microorganisms11081911 PMC10459532

[ece311670-bib-0013] Consortium, T. P. W. S. , & Ching, N. L. (2021). *Wolbachia*‐mediated sterility suppresses *Aedes aegypti* populations in the urban tropics. *medRxiv* . 10.1101/2021.06.16.21257922

[ece311670-bib-0014] Coon, K. L. , Brown, M. R. , & Strand, M. R. (2016). Mosquitoes host communities of bacteria that are essential for development but vary greatly between local habitats. Molecular Ecology, 25(22), 5806–5826. 27718295 10.1111/mec.13877 PMC5118126

[ece311670-bib-0015] Crawford, J. E. , Clarke, D. W. , Criswell, V. , Desnoyer, M. , Cornel, D. , Deegan, B. , Gong, K. , Hopkins, K. C. , Howell, P. , Hyde, J. S. , Livni, J. , Behling, C. , Benza, R. , Chen, W. , Dobson, K. L. , Eldershaw, C. , Greeley, D. , Han, Y. , Hughes, B. , … White, B. J. (2020). Efficient production of male *Wolbachia*‐infected *Aedes aegypti* mosquitoes enables large‐scale suppression of wild populations. Nature Biotechnology, 38(4), 482–492. 10.1038/s41587-020-0471-x 32265562

[ece311670-bib-0016] Czarnetzki, A. B. , & Tebbe, C. C. (2004). Detection and phylogenetic analysis of *Wolbachia* in Collembola. Environmental Microbiology, 6(1), 35–44. 14686939 10.1046/j.1462-2920.2003.00537.x

[ece311670-bib-0017] Duron, O. , Bernard, C. , Unal, S. , Berthomieu, A. , Berticat, C. , & Weill, M. (2006). Tracking factors modulating cytoplasmic incompatibilities in the mosquito *Culex pipiens* . Molecular Ecology, 15(10), 3061–3071. 10.1111/j.1365-294X.2006.02996.x 16911221

[ece311670-bib-0018] Endersby‐Harshman, N. M. , Ali, A. , Alhumrani, B. , Alkuriji, M. A. , Al‐Fageeh, M. B. , Al‐Malik, A. , Alsuabeyl, M. S. , Elfekih, S. , & Hoffmann, A. A. (2021). Voltage‐sensitive sodium channel (Vssc) mutations associated with pyrethroid insecticide resistance in *Aedes aegypti* (L.) from two districts of Jeddah, Kingdom of Saudi Arabia: Baseline information for a *Wolbachia* release program. Parasites & Vectors, 14(1), 361. 10.1186/s13071-021-04867-3 34247634 PMC8273952

[ece311670-bib-0019] Giger, J. N. , Hosch, S. , von Planta, N. G. , Bela, N. R. , Fuseini, G. , Oyana, R. N. N. , Houag, M. L. , Efiri, P. B. E. , Maye, V. O. N. , & García, G. A. (2024). Molecular evidence of high prevalence of *Wolbachia* species in wild‐caught *Aedes albopictus* and *Aedes aegypti* mosquitoes on Bioko Island, Equatorial Guinea. *Research Square* . 10.21203/rs.3.rs-4046525/v1

[ece311670-bib-0020] Gloria‐Soria, A. , Chiodo, T. G. , & Powell, J. R. (2018). Lack of evidence for natural *Wolbachia* infections in *Aedes aegypti* (Diptera: Culicidae). Journal of Medical Entomology, 55(5), 1354–1356. 29901734 10.1093/jme/tjy084 PMC6113644

[ece311670-bib-0021] Gu, X. , Ross, P. A. , Rodriguez‐Andres, J. , Robinson, K. L. , Yang, Q. , Lau, M. J. , & Hoffmann, A. A. (2022). A *w*Mel *Wolbachia* variant in *Aedes aegypti* from field‐collected *Drosophila melanogaster* with increased phenotypic stability under heat stress. Environmental Microbiology, 24(4), 2119–2135. 35319146 10.1111/1462-2920.15966 PMC9544352

[ece311670-bib-0022] Hegde, S. , Khanipov, K. , Albayrak, L. , Golovko, G. , Pimenova, M. , Saldaña, M. A. , Rojas, M. M. , Hornett, E. A. , Motl, G. C. , & Fredregill, C. L. (2018). Microbiome interaction networks and community structure from laboratory‐reared and field‐collected *Aedes aegypti*, *Aedes albopictus*, and *Culex quinquefasciatus* mosquito vectors. Frontiers in Microbiology, 9, 2160. 30250462 10.3389/fmicb.2018.02160 PMC6140713

[ece311670-bib-0023] Hernández, A. M. , Alcaraz, L. D. , Hernández‐Álvarez, C. , Romero, M. F. , Jara‐Servín, A. , Barajas, H. , Ramírez, C. M. , & Peimbert, M. (2024). Revealing the microbiome diversity and biocontrol potential of field *Aedes* ssp.: Implications for disease vector management. PLoS One, 19(4), e0302328. 10.1371/journal.pone.0302328 38683843 PMC11057774

[ece311670-bib-0024] Hoffmann, A. , & Turelli, M. (1997). Cytoplasmic incompatibility in insects. In S. L. O'Neill , A. A. Hoffmann , & J. H. Werren (Eds.), Influential passengers: Inherited microorganisms and invertebrate reproduction. Oxford University Press.

[ece311670-bib-0025] Hoffmann, A. A. , Ahmad, N. W. , Keong, W. M. , Ling, C. Y. , Ahmad, N. A. , Golding, N. , Tierney, N. , Jelip, J. , Putit, P. W. , Mokhtar, N. , Sandhu, S. S. , Ming, L. S. , Khairuddin, K. , Denim, K. , Rosli, N. M. , Shahar, H. , Omar, T. , Ridhuan Ghazali, M. K. , Aqmar Mohd Zabari, N. Z. , … Sinkins, S. P. (2024). Introduction of *Aedes aegypti* mosquitoes carrying *w*AlbB *Wolbachia* sharply decreases dengue incidence in disease hotspots. iScience, 27(2), 108942. 10.1016/j.isci.2024.108942 38327789 PMC10847733

[ece311670-bib-0026] Hoffmann, A. A. , Montgomery, B. , Popovici, J. , Iturbe‐Ormaetxe, I. , Johnson, P. , Muzzi, F. , Greenfield, M. , Durkan, M. , Leong, Y. , & Dong, Y. (2011). Successful establishment of *Wolbachia* in *Aedes* populations to suppress dengue transmission. Nature, 476(7361), 454–457. 21866160 10.1038/nature10356

[ece311670-bib-0027] Indriani, C. , Tanamas, S. K. , Khasanah, U. , Ansari, M. R. , Rubangi , Tantowijoyo, W. , Ahmad, R. A. , Dufault, S. M. , Jewell, N. P. , & Utarini, A. (2023). Impact of randomised *w*Mel *Wolbachia* deployments on notified dengue cases and insecticide fogging for dengue control in Yogyakarta City. Global Health Action, 16(1), 2166650. 36700745 10.1080/16549716.2023.2166650 PMC9894080

[ece311670-bib-0028] Kriesner, P. , Hoffmann, A. A. , Lee, S. F. , Turelli, M. , & Weeks, A. R. (2013). Rapid sequential spread of two *Wolbachia* variants in *Drosophila simulans* . PLoS Pathogens, 9(9), e1003607. 24068927 10.1371/journal.ppat.1003607 PMC3771877

[ece311670-bib-0029] Kulkarni, A. , Yu, W. , Jiang, J. , Sanchez, C. , Karna, A. K. , Martinez, K. J. , Hanley, K. A. , Buenemann, M. , Hansen, I. A. , & Xue, R. d. (2019). *Wolbachia pipientis* occurs in *Aedes aegypti* populations in New Mexico and Florida, USA. Ecology and Evolution, 9(10), 6148–6156. 31161026 10.1002/ece3.5198 PMC6540660

[ece311670-bib-0030] Kumar, N. P. , Kalimuthu, M. , Kumar, M. S. , Govindrajan, R. , Venkatesh, A. , Paramasivan, R. , Kumar, A. , & Gupta, B. (2022). Morphological and molecular characterization of *Aedes aegypti* variant collected from Tamil Nadu, India. Journal of Vector Borne Diseases, 59(1), 22–28. 10.4103/0972-9062.331413 35708400

[ece311670-bib-0031] Lambrechts, L. , Scott, T. W. , & Gubler, D. J. (2010). Consequences of the expanding global distribution of *Aedes albopictus* for dengue virus transmission. PLoS Neglected Tropical Diseases, 4(5), e646. 20520794 10.1371/journal.pntd.0000646 PMC2876112

[ece311670-bib-0032] Li, C. , Liu, S. , Zhou, H. , Zhu, W. , Cui, M. , Li, J. , Wang, J. , Liu, J. , Zhu, J. , Li, W. , Bi, Y. , Carr Michael, J. , Holmes Edward, C. , & Shi, W. (2023). Metatranscriptomic sequencing reveals host species as an important factor shaping the mosquito virome. Microbiology Spectrum, 11(2), e04655‐04622. 10.1128/spectrum.04655-22 36786616 PMC10101097

[ece311670-bib-0033] Liu, W. L. , Yu, H. Y. , Chen, Y. X. , Chen, B. Y. , Leaw, S. N. , Lin, C. H. , Su, M. P. , Tsai, L. S. , Chen, Y. , Shiao, S. H. , Xi, Z. Y. , Jang, A. C. C. , & Chen, C. H. (2022). Lab‐scale characterization and semi‐field trials of *Wolbachia* strain *w*AlbB in a Taiwan *Wolbachia* introgressed *ae*. *Aegypti* strain. PLoS Neglected Tropical Diseases, 16(1), e0010084. 10.1371/journal.pntd.0010084 35015769 PMC8752028

[ece311670-bib-0034] Martinez, J. , Ross, P. A. , Gu, X. , Ant, T. H. , Murdochy, S. M. , Tong, L. , da Silva Filipe, A. , Hoffmann, A. A. , & Sinkins, S. P. (2022). Genomic and phenotypic comparisons reveal distinct variants of *Wolbachia* strain *w*AlbB. Applied and Environmental Microbiology, 88(22), e01412‐01422. 36318064 10.1128/aem.01412-22 PMC9680635

[ece311670-bib-0035] Moreira, L. A. , Iturbe‐Ormaetxe, I. , Jeffery, J. A. , Lu, G. , Pyke, A. T. , Hedges, L. M. , Rocha, B. C. , Hall‐Mendelin, S. , Day, A. , Riegler, M. , Hugo, L. E. , Johnson, K. N. , Kay, B. H. , McGraw, E. A. , van den Hurk, A. F. , Ryan, P. A. , & O'Neill, S. L. (2009). A *Wolbachia* symbiont in *Aedes aegypti* limits infection with dengue, chikungunya, and *plasmodium* . Cell, 139(7), 1268–1278. 10.1016/j.cell.2009.11.042 20064373

[ece311670-bib-0036] Muharromah, A. F. , Reyes, J. I. L. , Kagia, N. , & Watanabe, K. (2023). Genome‐wide detection of *Wolbachia* in natural *Aedes aegypti* populations using ddRAD‐Seq. Frontiers in Cellular and Infection Microbiology, 13, 1252656. 10.3389/fcimb.2023.1252656 38162582 PMC10755911

[ece311670-bib-0037] Nazni, W. A. , Hoffmann, A. A. , NoorAfizah, A. , Cheong, Y. L. , Mancini, M. V. , Golding, N. , Kamarul, G. M. , Arif, M. A. , Thohir, H. , & NurSyamimi, H. (2019). Establishment of Wolbachia strain *w*AlbB in Malaysian populations of *Aedes aegypti* for dengue control. Current Biology, 29(24), 4241–4248.e4245. 31761702 10.1016/j.cub.2019.11.007 PMC6926472

[ece311670-bib-0038] Nikoh, N. , Tanaka, K. , Shibata, F. , Kondo, N. , Hizume, M. , Shimada, M. , & Fukatsu, T. (2008). *Wolbachia* genome integrated in an insect chromosome: Evolution and fate of laterally transferred endosymbiont genes. Genome Research, 18(2), 272–280. 18073380 10.1101/gr.7144908 PMC2203625

[ece311670-bib-0039] Pagendam, D. , Elfekih, S. , Nassar, M. S. , Nelson, S. , Almalik, A. M. , Tawfik, E. A. , Al‐Fageeh, M. B. , & Hoffmann, A. A. (2022). Spatio‐temporal modelling informing *Wolbachia* replacement releases in a low rainfall climate. Insects, 13(10), 949. 36292897 10.3390/insects13100949 PMC9604250

[ece311670-bib-0040] Regilme, M. A. F. , Inukai, T. , & Watanabe, K. (2021). Detection and phylogeny of *Wolbachia* in field‐collected *Aedes albopictus* and *Aedes aegypti* from Manila City, Philippines. *bioRxiv* . 10.1101/2021.08.24.457456

[ece311670-bib-0041] Reyes, J. I. L. , Suzuki, T. , Suzuki, Y. , & Watanabe, K. (2024). Detection and quantification of natural *Wolbachia* in *Aedes aegypti* in metropolitan Manila, Philippines using locally designed primers. Frontiers in Cellular and Infection Microbiology, 14, 1360438. 38562961 10.3389/fcimb.2024.1360438 PMC10982481

[ece311670-bib-0042] Rodpai, R. , Boonroumkaew, P. , Sadaow, L. , Sanpool, O. , Janwan, P. , Thanchomnang, T. , Intapan, P. M. , & Maleewong, W. (2023). Microbiome composition and microbial community structure in mosquito vectors *Aedes aegypti* and *Aedes albopictus* in northeastern Thailand, a dengue‐endemic area. Insects, 14(2), 184. https://www.mdpi.com/2075‐4450/14/2/184 36835753 10.3390/insects14020184 PMC9961164

[ece311670-bib-0043] Roslan, M. A. , Ngui, R. , Abd Karim, M.‐A.‐A. , Rosmini, U. S. , Ong, P. S. , Ahmad, M. A. , Lim, Y. A. L. , & Wan Sulaiman, W. Y. (2024). A study on *Wolbachia*‐dengue‐carrying *Aedes* mosquitoes (diptera: Culicidae) focuses on the sustainability and frequency of *Wolbachia* in high‐rise buildings in Selangor, Malaysia. Applied Entomology and Zoology. 10.1007/s13355-024-00870-z

[ece311670-bib-0044] Ross, P. A. , Axford, J. K. , Callahan, A. G. , Richardson, K. M. , & Hoffmann, A. A. (2020). Persistent deleterious effects of a deleterious *Wolbachia* infection. PLoS Neglected Tropical Diseases, 14(4), e0008204. 32243448 10.1371/journal.pntd.0008204 PMC7159649

[ece311670-bib-0045] Ross, P. A. , Callahan, A. G. , Yang, Q. , Jasper, M. , Arif, M. A. , Afizah, A. N. , Nazni, W. A. , & Hoffmann, A. A. (2020). An elusive endosymbiont: Does *Wolbachia* occur naturally in *Aedes aegypti*? Ecology and Evolution, 10(3), 1581–1591. 32076535 10.1002/ece3.6012 PMC7029055

[ece311670-bib-0046] Ross, P. A. , Elfekih, S. , Collier, S. , Klein, M. J. , Lee, S. S. , Dunn, M. , Jackson, S. , Zhang, Y. , Axford, J. K. , & Gu, X. (2023). Developing *Wolbachia*‐based disease interventions for an extreme environment. PLoS Pathogens, 19(1), e1011117. 36719928 10.1371/journal.ppat.1011117 PMC9917306

[ece311670-bib-0047] Ryan, P. A. , Turley, A. P. , Wilson, G. , Hurst, T. P. , Retzki, K. , Brown‐Kenyon, J. , Hodgson, L. , Kenny, N. , Cook, H. , & Montgomery, B. L. (2019). Establishment of *w*Mel *Wolbachia* in *Aedes aegypti* mosquitoes and reduction of local dengue transmission in Cairns and surrounding locations in northern Queensland, Australia. Gates Open Research, 3, 1547. 31667465 10.12688/gatesopenres.13061.1 PMC6801363

[ece311670-bib-0048] Sarwar, M. S. , Jahan, N. , Ali, A. , Yousaf, H. K. , & Munzoor, I. (2022). Establishment of *Wolbachia* infection in *Aedes aegypti* from Pakistan via embryonic microinjection and semi‐field evaluation of general fitness of resultant mosquito population. Parasites & Vectors, 15(1), 191. 10.1186/s13071-022-05317-4 35668540 PMC9169386

[ece311670-bib-0049] Sawadogo, S. P. , Kabore, D. A. , Tibiri, E. B. , Hughes, A. , Gnankine, O. , Quek, S. , Diabaté, A. , Ranson, H. , Hughes, G. L. , & Dabiré, R. K. (2022). Lack of robust evidence for a *Wolbachia* infection in *Anopheles gambiae* from Burkina Faso. Medical and Veterinary Entomology, 36(3), 301–308. 35876244 10.1111/mve.12601 PMC10053554

[ece311670-bib-0050] Somia, E. S. , Ullah, I. , Alyahya, H. S. , & Mahyoub, J. A. (2023). Identification of *Wolbachia* new strains from *Aedes aegypti* mosquitoes, the vector of dengue fever in Jeddah Province. BMC Microbiology, 23(1), 287. 37803282 10.1186/s12866-023-03010-9 PMC10557223

[ece311670-bib-0051] Surasiang, T. , Chumkiew, S. , Martviset, P. , Chantree, P. , & Jamklang, M. (2022). Mosquito larva distribution and natural *Wolbachia* infection in campus areas of Nakhon Ratchasima, Thailand. Asian Pacific Journal of Tropical Medicine, 15(7), 314–321. 10.4103/1995-7645.351763

[ece311670-bib-0052] Teo, C. , Lim, P. , Voon, K. , & Mak, J. (2017). Detection of dengue viruses and Wolbachia in *Aedes aegypti* and *Aedes albopictus* larvae from four urban localities in Kuala Lumpur, Malaysia. Tropical Biomedicine, 34(3), 583–597. 33592927

[ece311670-bib-0053] Thongsripong, P. , Chandler, J. A. , Green, A. B. , Kittayapong, P. , Wilcox, B. A. , Kapan, D. D. , & Bennett, S. N. (2018). Mosquito vector‐associated microbiota: Metabarcoding bacteria and eukaryotic symbionts across habitat types in Thailand endemic for dengue and other arthropod‐borne diseases. Ecology and Evolution, 8(2), 1352–1368. 29375803 10.1002/ece3.3676 PMC5773340

[ece311670-bib-0054] Tripet, F. , Lounibos, L. P. , Robbins, D. , Moran, J. , Nishimura, N. , & Blosser, E. M. (2011). Competitive reduction by satyrization? Evidence for interspecific mating in nature and asymmetric reproductive competition between invasive mosquito vectors. The American Journal of Tropical Medicine and Hygiene, 85(2), 265–270. 21813845 10.4269/ajtmh.2011.10-0677 PMC3144823

[ece311670-bib-0055] Vega‐Rúa, A. , Zouache, K. , Girod, R. , Failloux, A.‐B. , & Lourenço‐de‐Oliveira, R. (2014). High level of vector competence of *Aedes aegypti* and *Aedes albopictus* from ten American countries as a crucial factor in the spread of chikungunya virus. Journal of Virology, 88(11), 6294–6306. 24672026 10.1128/JVI.00370-14 PMC4093877

[ece311670-bib-0056] Větrovský, T. , & Baldrian, P. (2013). The variability of the 16S rRNA gene in bacterial genomes and its consequences for bacterial community analyses. PLoS One, 8(2), e57923. 23460914 10.1371/journal.pone.0057923 PMC3583900

[ece311670-bib-0057] Vinayagam, S. , Nirmolia, T. , Chetry, S. , Kumar, N. P. , Saini, P. , Bhattacharyya, D. R. , Bhowmick, I. P. , Sattu, K. , & Patgiri, S. J. (2023). Molecular evidence of *Wolbachia* species in wild‐caught *Aedes albopictus* and *Aedes aegypti* mosquitoes in four states of northeast India. Journal of Tropical Medicine, 2023, 6678627. 10.1155/2023/6678627 37706052 PMC10497363

[ece311670-bib-0058] Walker, T. , Quek, S. , Jeffries, C. L. , Bandibabone, J. , Dhokiya, V. , Bamou, R. , Kristan, M. , Messenger, L. A. , Gidley, A. , & Hornett, E. A. (2021). Stable high‐density and maternally inherited *Wolbachia* infections in *Anopheles moucheti* and *Anopheles demeilloni* mosquitoes. Current Biology, 31(11), 2310–2320.e2315. 33857432 10.1016/j.cub.2021.03.056 PMC8210651

[ece311670-bib-0059] Wijegunawardana, N. , Gunawardene, Y. S. , Abeyewickreme, W. , Chandrasena, T. , Thayanukul, P. , & Kittayapong, P. (2024). Diversity of *Wolbachia* infections in Sri Lankan mosquitoes with a new record of *Wolbachia* Supergroup B infecting *Aedes aegypti* vector populations. Scientific Reports, 14(1), 11966. 38796552 10.1038/s41598-024-62476-3 PMC11127934

[ece311670-bib-0060] Wong, M. L. , Liew, J. W. K. , Wong, W. K. , Pramasivan, S. , Mohamed Hassan, N. , Wan Sulaiman, W. Y. , Jeyaprakasam, N. K. , Leong, C. S. , Low, V. L. , & Vythilingam, I. (2020). Natural *Wolbachia* infection in field‐collected *Anopheles* and other mosquito species from Malaysia. Parasites & Vectors, 13, 1–15. 32787974 10.1186/s13071-020-04277-x PMC7425011

[ece311670-bib-0061] Yang, Q. , Chung, J. , Robinson, K. L. , Schmidt, T. L. , Ross, P. A. , Liang, J. , & Hoffmann, A. A. (2022). Sex‐specific distribution and classification of *Wolbachia* infections and mitochondrial DNA haplogroups in *Aedes albopictus* from the indo‐Pacific. PLoS Neglected Tropical Diseases, 16(4), e0010139. 35417447 10.1371/journal.pntd.0010139 PMC9037918

[ece311670-bib-0062] Zhang, H. , Gao, J. , Ma, Z. , Liu, Y. , Wang, G. , Liu, Q. , Du, Y. , Xing, D. , Li, C. , & Zhao, T. (2022). *Wolbachia* infection in field‐collected *Aedes aegypti* in Yunnan Province, southwestern China. Frontiers in Cellular and Infection Microbiology, 12, 1082809. 36530420 10.3389/fcimb.2022.1082809 PMC9748079

[ece311670-bib-0063] Zheng, X. , Zhang, D. , Li, Y. , Yang, C. , Wu, Y. , Liang, X. , Liang, Y. , Pan, X. , Hu, L. , & Sun, Q. (2019). Incompatible and sterile insect techniques combined eliminate mosquitoes. Nature, 572(7767), 56–61. 31316207 10.1038/s41586-019-1407-9

[ece311670-bib-0064] Zouache, K. , Martin, E. , Rahola, N. , Gangue, M. F. , Minard, G. , Dubost, A. , Van, V. T. , Dickson, L. , Ayala, D. , Lambrechts, L. , & Moro, C. V. (2022). Larval habitat determines the bacterial and fungal microbiota of the mosquito vector *Aedes aegypti* . FEMS Microbiology Ecology, 98(1), 1–11. 10.1093/femsec/fiac016 35147188

